# Pretreatment with Panaxatriol Saponin Attenuates Mitochondrial Apoptosis and Oxidative Stress to Facilitate Treatment of Myocardial Ischemia-Reperfusion Injury via the Regulation of Keap1/Nrf2 Activity

**DOI:** 10.1155/2022/9626703

**Published:** 2022-05-27

**Authors:** Huan Yao, Qian Xie, Qingman He, Lei Zeng, Jing Long, Yuanyuan Gong, Xueping Li, Xueping Li, Weiwei Liu, Zhiyi Xu, Huihui Wu, Chuan Zheng, Yongxiang Gao

**Affiliations:** ^1^Hospital of Chengdu University of Traditional Chinese Medicine, Chengdu, 610072 Sichuan, China; ^2^Basic Medical College, Chengdu University of Traditional Chinese Medicine, Chengdu, 611137 Sichuan, China; ^3^Chengdu Huasun Technology Group Inc., Ltd, Chengdu, 611731 Sichuan, China; ^4^National Engineering Research Center for Biomaterials, Chengdu, 610064 Sichuan, China; ^5^International Education College, Chengdu University of Traditional Chinese Medicine, Chengdu, 610075 Sichuan, China

## Abstract

Myocardial ischemia-reperfusion injury (MIRI) is a type of severe injury to the ischemic myocardium that can occur following recovery of blood flow, and for which, there is no effective treatment. Panaxatriol saponin (PTS), a major active component of *P. notoginseng*, has been used clinically to treat ischemia-related encephalopathy due to its antioxidant activity, but its effect on ischemic cardiomyopathy and underlying mechanism of action is still unclear. This study was performed to investigate the protective effect of PTS against MIRI and explore the potential underlying mechanisms. Hydrogen peroxide (H_2_O_2_) was used to stimulate cardiomyocytes, to mimic MIRI *in vitro*. Cell viability was tested using the CCK-8 method. The antioxidant activity of PTS in the H9c2 rat cardiomyocyte cell line was examined using 2′,7′-dichlorodihydrofluorescein diacetate (DCFH-DA). The levels of superoxide dismutase-1 (SOD1), SOD2, and heme oxygenase (HO-1) were determined by Western blotting and/or immunofluorescence. The antiapoptotic effect of PTS was determined. In addition, mitochondrial permeability transition pore (mPTP) opening and mitochondrial membrane potential (ΔΨm) changes were assessed. Changes in Keap1/Nrf2 activation were evaluated by Western blotting analysis, molecular docking, and immunoprecipitation. An *in vivo* MIRI model was established in rats, and the myocardial infarct size was measured by 2,3,5-triphenyltetrazolium chloride (TTC) staining. Myocardial enzyme activities were determined by ELISA or biochemical analyses. Furthermore, changes in Nrf2 activation were evaluated, and the regulatory effect of PTS on cardiomyocyte apoptosis was examined using the Nrf2 blocker, ML385. The results showed that PTS ameliorated the cardiomyocyte injury induced by H_2_O_2_, characterized by increased cell viability, decreased reactive oxygen species (ROS) production, and promotion of SOD1, SOD2, and HO1 expression. PTS inhibited cardiomyocyte apoptosis *in vivo* and *in vitro*. PTS also reduced mPTP opening and stabilized ΔΨm in H9c2 cells. Molecular docking and immunoprecipitation study revealed that PTS can disrupt Keap1/Nrf2 interaction by directly blocking the binding site of Nrf2 in the Keap1 protein. *In vivo*, PTS decreased the area of myocardial infarction and attenuated pathological damage in ischemia-reperfusion (I/R) rats. In addition, the activities of myocardial injury markers were decreased by PTS. Finally, PTS regulated nuclear translocation of Nrf2, and ML385 blocked the therapeutic effect of PTS *in vivo* and *in vitro*. These results suggested that PTS has therapeutic potential for MIRI by targeting Keap1/Nrf2 activity.

## 1. Introduction

Coronary heart disease (CHD) is the leading cause of death and disability worldwide [1] and is usually attributable to the detrimental effects of acute myocardial ischemia-reperfusion injury (MIRI) [2]. MIRI usually occurs in patients with acute ST-segment elevation myocardial infarction (STEMI). In these patients, the most effective therapeutic intervention to reduce acute myocardial ischemic injury and limit the scale of myocardial infarction is timely and effective myocardial reperfusion via primary percutaneous coronary intervention (PPCI) or thrombolytic therapy [2]. However, myocardial reperfusion itself induces further death of myocardial cells.

Several mechanisms have been proposed to contribute to reperfusion injury. It is generally accepted that oxidative stress is a major contributor to the onset and development of MIRI [3, 4]. Oxidative stress triggered by excessive reactive oxygen species (ROS) is considered an essential initiator of cardiomyocyte apoptosis. Therefore, antiapoptotic treatments represent a promising research direction for the development of therapeutic strategies for ischemic cardiomyopathy; it is possible to control the disease process and protect the functional reserve of the myocardium [5–7].

Mitochondrial dysfunction has been reported to be closely related to ischemic heart disease [8–10]. Under conditions of myocardial ischemia and hypoxia, mitochondria mainly achieve energy metabolism through anaerobic glycolysis, which leads to the production of large amounts of lactic acid; this causes intracellular acidosis, which in turn damages mitochondria. During myocardial reperfusion, due to the instantaneous increase in local blood oxygen concentration, ROS bursts occur in mitochondria and further undermine the dynamic balance between oxidation and reduction, leading to mitochondrial permeability transition pore (mPTP) opening, abnormal distribution of charged ions inside and outside the membranes, and disruption of electrochemical gradients, thus causing mitochondrial membrane potential (ΔΨm) loss.

Numerous natural plants have been reported to exhibit antioxidant activity, and recent studies have indicated their therapeutic potential for ischemic cardiomyopathy, including MIRI [11–14]. *Panax notoginseng*, the root of *Panax notoginseng* (Burk.) F.H. Chen, has been used as a traditional herbal medicine in China for more than 600 years due to its beneficial effects in preventing and treating various diseases, including cardiovascular and cerebrovascular diseases [15]. Panaxatriol saponin (PTS), one of the major effective components of *P. notoginseng*, has been used clinically in China for the treatment of cerebral diseases due to its antioxidant, anti-inflammatory, antiplatelet, and angiogenesis-promoting activities [16–19]. Furthermore, an early study indicated that pretreatment with ginseng total saponin ameliorated ischemia-reperfusion (I/R)-induced myocardial damage, and this protective effect was mediated by a decrease in oxidative stress [20]. However, the specific effects of PTS on MIRI, and the underlying mechanism of action, remain largely unknown.

The present study was performed to investigate the protective effect of PTS on MIRI, and to identify the potential mechanisms underlying these effects. The results indicated that PTS pretreatment attenuated oxidative stress-induced cardiomyocyte apoptosis and protected the heart against MIRI. The cardioprotective effects of PTS involved reinforcement of the antioxidant system via the activation of Nrf2.

### 1.1. Reagents

PTS extracted from *P. notoginseng* was obtained from Huasun Group Co., Ltd. (Sichuan, China). The main components of PTS were the ginsenoside Rg1, notoginsenoside R1, and ginsenoside Re (Supplementary Materials: Figure S1).

### 1.2. Animals and Experimental Groups

Adult male Sprague–Dawley rats (8–10 weeks old, 180–200 g) were provided by Chengdu Dashuo Experimental Animal Center. All animals used in the present study received ethical and humane care. Experimental procedures were conducted in compliance with the National Institutes of Health Guidelines for Care and Use of Laboratory Animals and were approved by the Bioethics Committee of Chengdu University of Traditional Chinese Medicine.

The animal experiments had two stages. In the first stage, a total of 60 rats were divided into the following six groups (*n* = 10 per group) to determine the optimal concentration of PTS: I/R group, 60 min of ischemia followed by 24 h of reperfusion; sham group, the same operation as the I/R group, but the left anterior descending (LAD) branch was not ligated; sham+PTS group, intragastric administration of 100 mg/kg/d PTS aqueous solution and the same operation as in the sham group; I/R+PTS groups, intragastric administration of 25, 50, or 100 mg/kg/d PTS aqueous solution followed by I/R operation. The pretreatment was carried over 7 days. The sham and I/R groups were pretreated with vehicle (water) before the operation. In the second stage, a total of 50 rats were divided into five groups (*n* = 10 per group) to explore the underlying mechanisms: I/R group, same as the first stage; sham group, same as the first stage; sham+PTS group, same as the first stage; I/R+PTS group, treatment with the optimal concentration of PTS determined in the first stage, and I/R as in the first stage; PTS+ML385 group, treatment with the optimal concentration of PTS 30 min after intraperitoneal injection of ML385 at a dose of 30 mg/kg [21].

### 1.3. Establishment of the Rat Myocardial I/R Injury Model

The rat myocardial I/R injury model was produced as described previously [22]. Briefly, rats were anesthetized by intraperitoneal injection of 3% sodium pentobarbital (50 mg/kg). The left ascending artery was occluded using a 7–0 silk suture tied transiently over PE-10 tubing for 60 min, after which the knot on the PE-10 tubing was cut. Successful ischemia was determined by the elevation of the ST segment on electrocardiography (ECG). Sham-operated control animals underwent the same surgical procedures, with the exception of LAD coronary artery occlusion.

### 1.4. Histological Analysis

Rat hearts were extracted and fixed in 4% paraformaldehyde in 0.1 mol/L phosphate-buffered saline (PBS) overnight, and embedded in paraffin. Sections (5 *μ*m thick) were collected and deparaffinized. Hematoxylin and eosin (H&E) staining was performed as described previously [23].

### 1.5. Immunofluorescence Analysis

For immunofluorescence analysis, sections or cells were incubated with primary antibodies at 4°C overnight. The next day, sections or cells were incubated with fluorescein-conjugated secondary antibody for 1 hour at room temperature. Finally, the sections were sealed with Slow Fade Gold antifade reagent with DAPI. Image-Pro Plus 6.0 (Media Cybernetics, Inc., Rockville, MD, USA) was used to analyze images and assess the results. The primary antibodies used for immunofluorescence analysis are listed in Supplementary Materials: Table S1.

### 1.6. Western Blotting Analysis and Immunoprecipitation (IP) Assay

Protein was extracted from heart tissue or cells using RIPA buffer and assessed using a BCA Protein Assay Kit (Beyotime, Jiangsu, China). The protein lysates were separated by 10% SDS-PAGE and transferred onto polyvinylidene difluoride (PVDF) membranes. The membranes were blocked with 5% skim milk in Tris-buffered saline (TBS) for 1 h and incubated with primary antibodies overnight at 4°C. The next day, after washing with Tris-buffered saline Tween-20 (TBST), the membranes were incubated with horseradish peroxidase (HRP)-conjugated secondary antibody for 1 h at room temperature.

For the IP assay, an Immunoprecipitation Kit with Protein A+G Magnetic Beads (Beyotime, Shanghai, China) was used. The main procedure was carried out in accordance with the manufacturer's instructions. Briefly, Keap1 or Nrf2 antibody (10 *μ*g) was incubated with magnetic beads (25 *μ*L) for 60 min. Cell lysates were mixed with antibody-coupled magnetic beads and incubated overnight at 4°C on a rotator. Precipitated proteins (60 *μ*L) were eluted from beads and prepared for Western blot analysis. Primary antibodies used for Western blotting analysis and IP are listed in Supplementary Materials: Table S1.

### 1.7. Cell Culture

The rat cardiomyocyte cell line, H9c2, was purchased from the Cell Bank of the Chinese Academy of Sciences (Shanghai, China) and cultured in Dulbecco's modified Eagle's medium (DMEM; HyClone, Logan, UT, USA) supplemented with 10% fetal bovine serum (FBS), 100 U/mL penicillin, and 100 *μ*g/mL streptomycin (HyClone), at 37°C in a humidified atmosphere containing 5% CO_2_.

### 1.8. Cell Counting Kit-8 Assay

Cell viability was examined by Cell Counting Kit (CCK)-8 assay (Biosharp, Hefei, China) in accordance with the manufacturer's instructions. Briefly, 3 × 10^3^ H9c2 cells were seeded in each well of a 96-well culture plate and treated with different concentrations of PTS or hydrogen peroxide (H_2_O_2_) at different time points. The absorbance of the sample at 450 nm was measured using a microplate reader.

### 1.9. Isolation and Culture of Neonatal Rat Primary Cardiomyocytes

Neonatal rat primary cardiomyocytes (NPCMs) were obtained from Sprague–Dawley rats at 48 ± 6 h after birth, as described previously [24]. Briefly, rats were euthanized by decapitation, and the heart was removed and placed in a culture dish containing Hank's balanced salt solution (HBSS) on ice. The hearts were digested with 1% type II collagenase and trypsin (2 : 1) without EDTA. After 1 h of natural sedimentation, the myocardial fibrocytes were discarded to obtain ventricular myocytes. The cells began to beat synchronously 1–2 days after plating, and the experiment was initiated. Cells were maintained in DMEM with 10% fetal bovine serum, 100 U/mL penicillin, and 100 *μ*g/mL streptomycin, and cultured at 37°C in an atmosphere of 5% CO_2_.

### 1.10. TUNEL Assay

Apoptosis was assessed by TUNEL assay in accordance with the manufacturer's instructions (Yeasen, Shanghai, China). Briefly, samples were fixed in 4% paraformaldehyde for 20 min, and then incubated in 0.1% Triton X-100 for 30 min, and covered with TUNEL reaction mixture. The samples were then incubated in a humidified chamber for 1 h at 37°C in the dark and then subjected to TUNEL staining. Finally, apoptotic cells were visualized using an inverted fluorescence microscope and counted in four randomly selected fields in each group.

### 1.11. Flow Cytometry

For flow cytometry, cells were harvested and stained with Annexin V-FITC and propidium iodide (PI) (Beyotime) for 30 min at room temperature. The cells were then washed twice with PBS, and the fluorescence data obtained from the cell population were analyzed with CellQuest software (BD Biosciences, Franklin Lakes, NJ, USA).

### 1.12. Measurement of Intracellular ROS Accumulation

2′,7′-Dichlorodihydrofluorescein diacetate (DCFH-DA; Yeasen) was used to determine the intracellular ROS level in H9c2 cells. Briefly, H9c2 cells were incubated with 10 *μ*M DCFH-DA in DMEM without FBS at 37°C for 20 minutes and then washed three times with DMEM. The 2′,7′-dichlorofluorescein (DCF) fluorescence was observed using an inverted fluorescence microscope.

### 1.13. Measurement of Myocardial Infarct Size

Following 24 h of reperfusion, myocardial infarct size (INF) was evaluated by 2,3,5-triphenyltetrazolium chloride (TTC) staining, as described previously [25]. Briefly, rats were killed and the hearts were excised. The left ventricle was frozen at −80°C for 10 min and then sectioned transversely into 2 mm thick slices and incubated in 1% TTC dissolved in PBS at 37°C for 8–10 min. Subsequently, sections were immersed in 4% formaldehyde for 12 h and photographed. Areas of INF were measured digitally using Image-Pro Plus 6.0 (Media Cybernetics, Inc., Bethesda, MD, USA). INF is expressed as a percentage of the left ventricular (LV) area (INF/LV).

### 1.14. Detection of Mitochondrial Permeability Transition Pore Opening

mPTP opening was detected using calcein AM (Beyotime) and MitoTracker Red CMXRos (Beyotime) in accordance with the manufacturer's instructions. Briefly, H9c2 cells were incubated with 200 nM MitoTracker Red CMXRos working solution at 37°C for 20 minutes and washed three times with PBS. For calcein AM staining, cells were incubated with 2 *μ*M calcein AM working solution at 37°C for 30 min. The working solution was then removed and replaced with fresh culture medium (DMEM supplemented with 10% FBS, 100 U/mL penicillin, and 100 *μ*g/mL streptomycin). Fluorescence was observed using an inverted fluorescence microscope.

### 1.15. Mitochondrial Membrane Potential Detection

The mitochondrial membrane potential (ΔΨm) of H9c2 cells was determined using a JC-1 Mitochondrial Membrane Potential Assay Kit (MCE, Monmouth Junction, NJ, USA) according to the manufacturer's instructions using a fluorescence microscope. The red fluorescence of JC-1 was detected at 590 nm with an excitation wavelength of 585 nm, and the green fluorescence was detected at 529 nm with an excitation wavelength of 514 nm.

### 1.16. ELISA

The rat serum levels of myoglobin (MB) and cardiac troponin-T (cTn-T) were determined using enzyme-linked immunosorbent assay (ELISA) kits according to the manufacturer's instructions (Mlbio, Shanghai, China).

### 1.17. Serum Biochemical Detection

The levels of creatine kinase (CK), CK-MB, and lactate dehydrogenase (LDH) in sera obtained by centrifugation of blood samples from rats were measured using an automatic biochemical analyzer (Mindray, Shenzhen, China).

### 1.18. Transmission Electron Microscopy

Transmission electron microscopy (TEM) was performed as described previously [25]. Briefly, cardiac tissue was dissected into 1 mm^3^ piece and fixed in 4% paraformaldehyde and 2% glutaraldehyde in 0.1 mol/L sodium cacodylate buffer (pH 7.2) overnight at 4°C. Following several washes in buffer, the samples were post-fixed with 2% osmium tetroxide and 1% uranyl acetate for 2 h, rinsed in water, dehydrated in an ascending ethanol series followed by 100% acetone, and then infiltrated and embedded in Eponate. Ultrathin sections were cut on a microtome and mounted onto 200-hex-mesh copper grids. The sections were exposed to the primary stain (5% aqueous uranyl acetate) followed by the secondary stain (lead citrate) and then visualized by TEM (H-600IV; Hitachi, Tokyo, Japan).

### 1.19. Molecular Docking

To acquire molecular insights on the binding mode of PTS in the Keap1 binding site, molecular docking studies were carried out. The crystal structure of Keap1 (entry: 4L7B) was downloaded from the RCSB Protein Data Bank (http://www.rcsb.org). Then, the Keap1 molecular was dehydrated and delete small-molecule ligands, and saved in PDB format. The two-dimensional structures of ginsenoside Rg1 (CID: 441923), notoginsenoside R1 (CID: 441934), and ginsenoside Re (CID: 441921) were downloaded from the PubChem database (https://pubchem.ncbi.nlm.nih.gov/). Python 3.8 was used to open RDKit software package to minimize the energy and add polar hydrogen of compounds. Thereafter, Smina software was used to dock Keap1 and PyMol 2.4 software was used to view the docking results.

### 1.20. Statistics

Quantitative data are presented as means ± standard error of the mean (SEM). Comparisons of multiple groups were determined by one-way ANOVA with Tukey's post hoc test using SPSS 21.0 (SPSS Inc., Chicago, USA). *P* values < 0.05 were considered indicative of statistical significance.

## 2. Results

### 2.1. Effects of PTS on H_2_O_2_-Induced H9c2 Cell Redox Homeostasis Disorders

**Figure 1:**
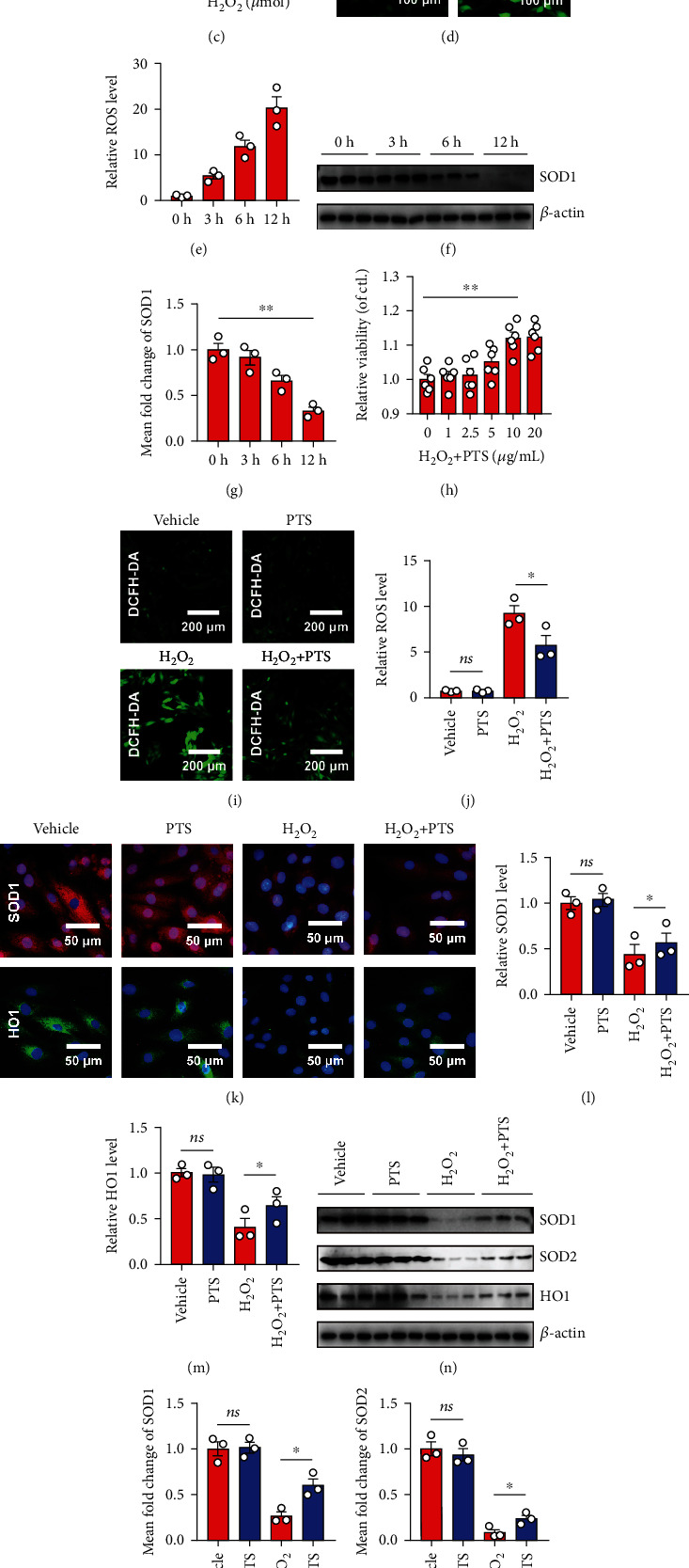
Effects of PTS on H_2_O_2_-induced H9c2 cell redox homeostasis disorders. H9c2 cells were treated with different concentrations of H_2_O_2_ (0, 50, 100, 200, 400, or 600 *μ*M) for 3, 6, or 12 hours. (a–c) The viability of H9c2 cells was determined by the CCK-8 assay. Values were normalized relative to the control group (representing 100% cell viability). Data are expressed as the mean ± SEM (*n* = 6). ^∗^*P* < 0.05, ^∗∗^*P* < 0.01. (d) Representative DCFH-DA images of H9c2 cells treated with 200 *μ*M H_2_O_2_ for 12 h. (e) Relative ROS levels were quantified using ImageJ software (NIH, Bethesda, MD, USA). (f) Representative Western blotting results of SOD1 in 200 *μ*M H_2_O_2_-treated H9c2 cells. (g) Relative SOD1 expression levels at various time points were quantified using ImageJ. H9c2 cells were preincubated with different concentrations of PTS (0, 1, 2.5, 5, 10, and 20 *μ*g/mL) for 12 h, followed by treatment with 200 *μ*M H_2_O_2_ for 12 h. (h) The viability of H9c2 cells was determined by CCK-8 assay. Values were normalized relative to the control group (representing 100% cell viability). (i) Representative DCFH-DA images of H9c2 cells. (j) Relative ROS levels were quantified using ImageJ. (k) Immunofluorescence assay was performed to determine the changes in SOD1 and HO1 protein levels in H9c2 cells. (l, m) SOD1 and HO1 levels were quantified using ImageJ. (n) Western blotting analysis showed the changes in SOD1, SOD2, and HO1 protein levels in H9c2 cells. (o–q) SOD1, SOD2, and HO1 levels were quantified using ImageJ. Data are expressed as the mean ± SEM (*n* = 3). ^∗^*P* < 0.05, ^∗∗^*P* < 0.01. ns: not significant; DCFH-DA: 2′,7′-dichlorodihydrofluorescein diacetate; PTS: panaxatriol saponins; SOD: superoxide dismutase.

Several mechanisms are involved in MIRI, particularly ROS overproduction. Previous studies have shown that using H_2_O_2_ to stimulate cardiomyocytes can mimic MIRI *in vitro* [26, 27]. To determine the ability of H_2_O_2_ to induce oxidative stress damage *in vitro*, H9c2 cells were selected and stimulated with different concentrations of H_2_O_2_ (0, 50, 100, 200, 400, and 600 *μ*M) for 3, 6, and 12 h, and cell viability was examined by CCK-8 assay. As shown in Figures 1(a)–1(c), compared with cells in the control group (0 *μ*M), H_2_O_2_ induced dose-dependent decreases in cell viability at 3, 6, and 12 h, with the maximum reduction seen at 12 h with 200 *μ*M H_2_O_2_. Therefore, this concentration was used in subsequent experiments. After exposure to H_2_O_2_, mean DCFH-DA fluorescence intensity increased significantly in a dose-dependent manner at 3, 6, and 12 h in H9c2 cells, indirectly confirming that H_2_O_2_ increases the generation of ROS in cardiomyocytes in a dose-dependent manner (Figures 1(d) and 1(e)). In addition, the activity of the antioxidant enzyme, superoxide dismutase-1 (SOD1), was significantly decreased after exposure to H_2_O_2_ (Figures 1(f) and 1(g)). Next, the antioxidant activities of PTS in H9c2 cells were examined. We first analyzed the effect of PTS on cell viability in the presence of H_2_O_2_ (200 *μ*M) and found that the reduction in cell viability was markedly ameliorated by pretreatment with PTS for 12 h at a concentration of 10 *μ*g/mL (Figure 1(h)). Therefore, this PTS concentration was chosen for subsequent experiments. To confirm that the effects of PTS on H9c2 cell survival were caused by ROS inhibition, the abovementioned experiments were performed in the presence of 10 *μ*g/mL PTS. H_2_O_2_ significantly increased the ROS components of the cells and impaired their antioxidant capacity, which was reflected in increased DCFH-DA fluorescence intensity and decreased SOD1 fluorescence intensity and protein expression. However, PTS pretreatment significantly ameliorated the dysregulation of antioxidant capacity of H9c2 cells, including downregulation of ROS (Figures 1(i) and 1(j)) but upregulation of SOD1, SOD2, and HO1 which are the major components of antioxidant enzyme (Figures 1(k)–1(q)). Taken together, these observations confirmed the ability of H_2_O_2_ to induce oxidative stress damage in cardiomyocytes and the therapeutic effect of PTS.

### 2.2. Effects of PTS on H_2_O_2_-Induced Apoptosis of H9c2 Cells and NRCMs

**Figure 2:**
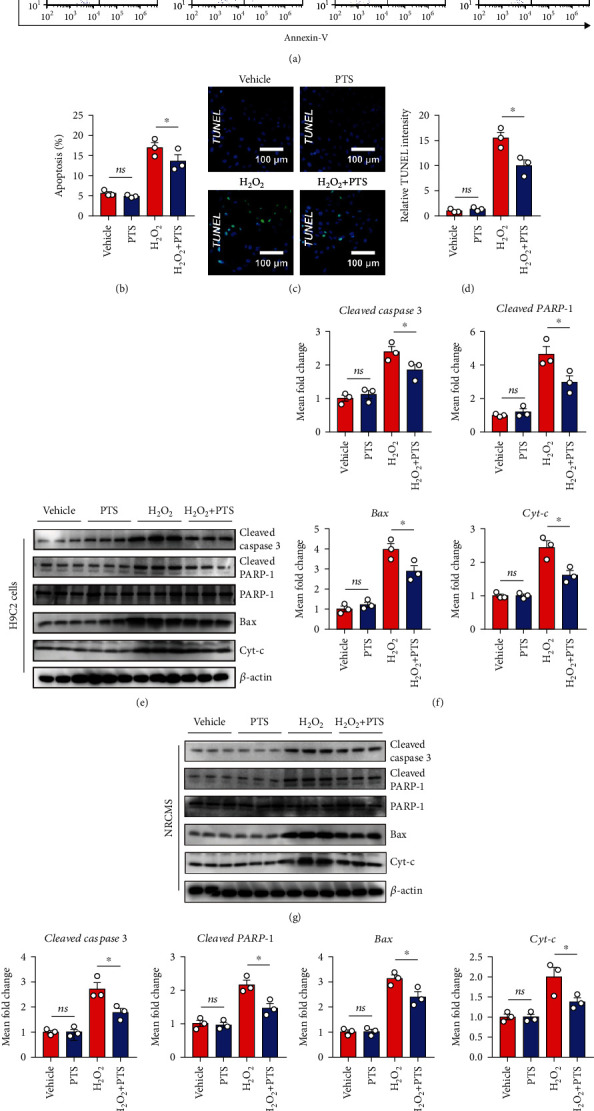
Effects of PTS on the H_2_O_2_-induced apoptosis of H9c2 cells and NRCMs. H9c2 cells were preincubated with PTS (10 *μ*g/mL) for 12 h followed by stimulation with 200 *μ*M H_2_O_2_ for 12 h. (a) The apoptosis ratio was measured by flow cytometry. (b) The results were quantified using ImageJ software. (c) Representative TUNEL staining results of H9c2 cells. (d) Relative TUNEL intensities of each group were quantified using ImageJ. (e) Western blotting analysis showed the changes in apoptosis-related protein expression in H9c2 cells. (f) Relative expression levels of cleaved caspase-3, cleaved PRAR-1, Bax, and Cyt-c were quantified using ImageJ. NRCMs were preincubated with PTS (10 *μ*g/mL) for 12 h followed by treatment with 200 *μ*M H_2_O_2_ for 12 h. (g) Western blotting analysis showed the changes in expression of apoptosis-related proteins of NRCMs. (h) Relative expression levels of cleaved caspase-3, cleaved PRAR-1, Bax, and Cyt-c were quantified using ImageJ. Data are expressed as the mean ± SEM (*n* = 3). ^∗^*P* < 0.05, ^∗∗^*P* < 0.01. ns: not significant; NRCMs: neonatal rat cardiomyocytes.

Cardiomyocyte apoptosis induced by oxidative stress injury is a key event in the pathogenesis of MIRI. To verify the effect of PTS on cardiomyocyte apoptosis *in vitro*, pretreated H9c2 cells were stained with Annexin V/PI and then detected by flow cytometry. After 12 hours of H_2_O_2_ treatment, the H9c2 cells underwent obvious apoptosis, i.e., there was a significant increase in the apoptosis rate. However, the apoptosis rate was lower in the H_2_O_2_+PTS than H_2_O_2_ group (Figures 2(a) and 2(b)). Similar results were obtained by TUNEL assay using an inverted fluorescence microscope, indicating that PTS ameliorated the marked elevation of TUNEL fluorescence intensity by H_2_O_2_ stimulation (Figures 2(c) and 2(d)). Moreover, the levels of apoptosis-related proteins, including cleaved caspase-3, cleaved PARP-1, Bax, and cytochrome-c (Cyt-c), were analyzed. Our data demonstrated that the levels of cleaved caspase-3, Bax, and cleaved PARP-1, as well as Cyt-c, were significantly higher in the H_2_O_2_ than vehicle and PTS alone groups at 12 h after treatment. However, the changes in these indicators compared with the H_2_O_2_ group were markedly ameliorated in the PTS+H_2_O_2_ group (Figures 2(e) and 2(f)) (Supplementary materials: Figure S2). Furthermore, similar results were obtained in NRCMs (Figures 2(g) and 2(h)). The above results suggested that PTS ameliorated H_2_O_2_-induced cardiomyocyte apoptosis.

### 2.3. Effects of PTS on mPTP Opening and Mitochondrial Membrane Potential (ΔΨm) of H9c2 Cells

**Figure 3:**
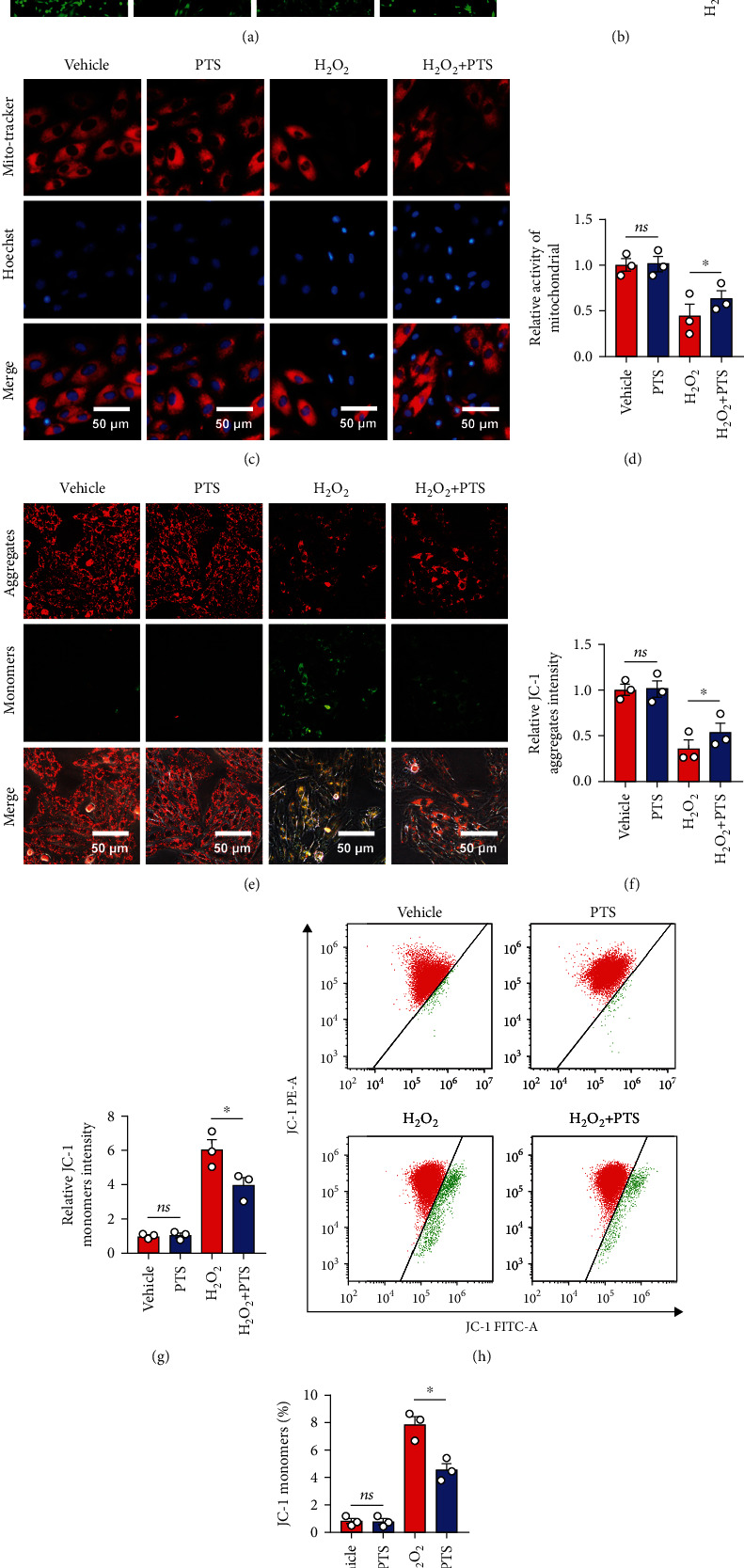
Effects of PTS on mPTP opening and the mitochondrial membrane potential (ΔΨm) of H9c2 cells. H9c2 cells were preincubated with PTS (10 *μ*g/mL) for 12 h followed by treatment with 200 *μ*M H_2_O_2_ for 12 h. (a) The representative images of calcein AM staining. (b) Relative fluorescence intensity of calcein AM. (c) The status of mitochondria in H9c2 cells was determined by MitoTracker Red CMXRos. (d) Relative mPTP opening was quantified using ImageJ software. (e) Effects of PTS on the ΔΨm of H9c2 cells based on JC-1 staining. (f, g) Quantitative analysis of ΔΨm using ImageJ. (h) After JC-1 staining, PTS-treated H9c2 cell populations were analyzed by flow cytometry. (i) The percentage of JC monomer-positive cells was quantified using ImageJ. Data are expressed as the mean ± SEM (*n* = 3). ^∗^*P* < 0.05, ^∗∗^*P* < 0.01. ns: not significant; mPTP: mitochondrial permeability transition pore.

Mitochondrial dysfunction is one of the main pathways of apoptosis and thus contributes to I/R injury. In the process of apoptosis, the permeability of the inner mitochondrial membrane increases, and the influx of Ca^2+^ leads to loss of the mitochondrial transmembrane potential. This change is mainly caused by the opening of mPTPs between the inner and outer mitochondrial membranes. Therefore, we investigated mPTP opening in H_2_O_2_-treated H9c2 cells by monitoring the fluorescence of mitochondrial-entrapped calcein using the calcein AM and CoCl_2_ coloading method [28]. As shown in Figures 3(a) and 3(b), the fluorescence intensity of mitochondrial calcein was significantly decreased in the H_2_O_2_ group compared with the vehicle group, indicating that mPTP opening was enhanced following H_2_O_2_ treatment. Surprisingly, pretreatment with PTS markedly increased the fluorescence intensity compared with the H_2_O_2_ group, suggesting that PTS inhibited H_2_O_2_-induced mPTP opening in H9c2 cells. Next, we used MitoTracker Red CMXRos and Hoechst costaining to directly observe mitochondrial activity, to determine the changes in ΔΨm in H9c2 cells. Similarly, the fluorescence intensity of MitoTracker Red CMXRos was significantly inhibited in apoptotic cells in the H_2_O_2_ group, indicating that the ΔΨm level increased following H_2_O_2_ treatment. In the H_2_O_2_+PTS group, however, fewer apoptotic H9c2 cells were observed, as indicated by bright Hoechst staining. PTS pretreatment also markedly increased the fluorescence intensity compared with the H_2_O_2_ group (Figures 3(c) and 3(d)), indicating that PTS inhibited H_2_O_2_-induced loss of ΔΨm in H9c2 cells. This conclusion was confirmed by subsequent assay. JC-1 staining showed that the H9c2 cells in the H_2_O_2_ group had low levels of red fluorescence and rich green fluorescence relative to the vehicle and PTS groups. Interestingly, H9c2 cells in the H_2_O_2_+PTS group exhibited attenuated green fluorescence and enhanced red fluorescence, as detected by fluorescence microscopy (Figures 3(e)–3(g)) and flow cytometry (Figures 3(h) and 3(i)). These results suggested that H_2_O_2_-induced mPTP opening and loss of ΔΨm in H9c2 cells were attenuated by PTS.

### 2.4. PTS Binds to the Kelch Domain of Keap1 to Regulate Nrf2 Activity

**Figure 4:**
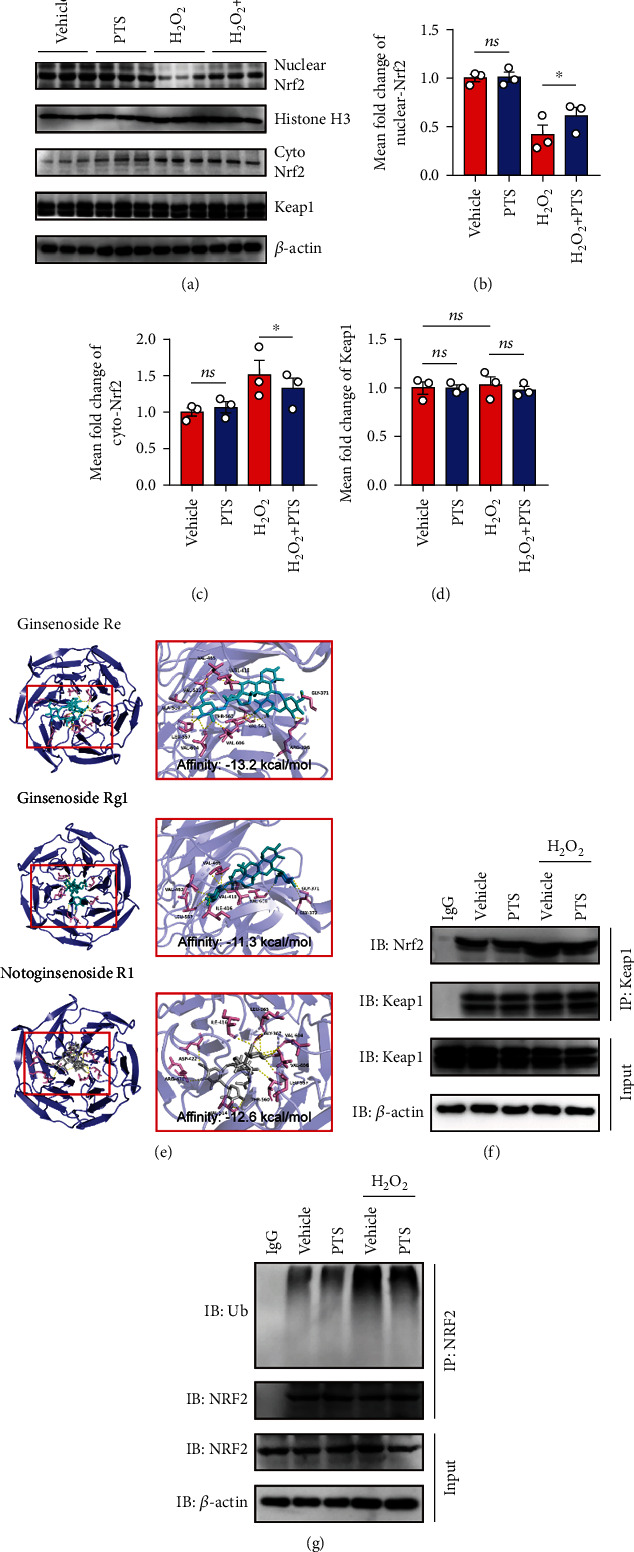
PTS binds to the Kelch domain of Keap1 to regulate Nrf2 activity. (a) Representative Western blotting analysis of Nrf-2 in the cytoplasm and nuclear fractions as well as Keap1 expression in whole-cell lysates. (b–d) Nuclear Nrf2, cytoplasm Nrf2, and Keap1 levels were quantified using ImageJ software. Data are expressed as the mean ± SEM (*n* = 3). ^∗^*P* < 0.05, ^∗∗^*P* < 0.01; ns: not significant. (e) *In silico* molecular docking of PTS with Keap1. A two-dimensional diagram shows the interactions of PTS to the amino acid residues in the binding pocket of Keap1. (f) Anti-Keap1 immunoprecipitation was analyzed by immunoblot with anti-Nrf2 antibodies for detection of Keap1-conjugated Nrf2. (g) Anti-Nrf2 immunoprecipitation was analyzed by immunoblot with anti-ubiquitin antibodies for detection of ubiquitin-conjugated Nrf2. Data are expressed as the mean ± SEM (*n* = 3). ^∗^*P* < 0.05, ^∗∗^*P* < 0.01; ns: not significant.

The Keap1/Nrf2 signaling is regarded as one critical endogenous antioxidative stress pathway discovered so far. Previous studies indicated that Nrf2 translocates from the cytoplasm to the nucleus, where it regulates the expression of antioxidant genes under conditions of stress [29]. To gain mechanistic insights into PTS-induced inhibition of oxidative stress *in vitro*, we next investigated the Keap1/Nrf2 signaling in H9c2 cells. We first analyzed Nrf2 levels in the cytoplasm and nucleus of H_2_O_2_-treated H9c2 cells. The results indicated that cytoplasmic Nrf2 level increased, and the nuclear Nrf2 level decreased, after H_2_O_2_ treatment. Conversely, PTS promoted Nrf2 nuclear translocation from the cytoplasm to the nucleus (Figures 4(a)–4(c)). Since these data revealed the antioxidant stress activity of PTS is probably associated with the regulation of the Nrf2 activity. And it has been well understood that Kelch-like ECH-associated protein-1 (Keap1) ubiquitinates Nrf2 to promote its degradation and is a negative regulator of Nrf2. We thus next determined the change of Keap1 protein in H9c2 cells. Impressively, the expression of Keap1 was not changed after stimulation of H_2_O_2_ and/or PTS compared with vehicle control (Figures 4(a) and 4(d)). Thus, we hypothesized that PTS may competitively inhibit the Nrf2-Keap1 interaction by binding to the active region of Keap-1. To confirm this conjecture, an in silico molecular docking study was performed with PTS on the crystal structure of Keap1 Kelch domain to determine if PTS can directly inhibit by interfering with interactions between Nrf2 and Keap1. Impressively, the molecular docking of PTS with Keap1 protein revealed that PTS binds to Kelch domain of Keap1 protein efficiently with the affinity of -13.2, -11.3, and -12.3 kcal/mol (Figure 4(e)). Because our data indicated that PTS binds to the Kelch domain of Keap1 protein, we hypothesize PTS may directly compete and restrict the binding of Nrf2 to Keap1 and thereby prevent its ubiquitination and subsequent degradation leading to activation of Nrf2. Thus, we performed immunoprecipitation assay to determine whether PTS restricts the binding of Nrf2 to Keap1and inhibited Nrf2 ubiquitination to regulate Nrf2 expression. As shown in Figures 4(f) and 4(g), anti-Keap1 immunoprecipitation was analyzed by immunoblot with anti-Nrf2 antibody for detection of Keap1-conjugated Nrf2. The results indicated that compared with vehicle treatment, PTS can block the interaction of Nrf2 and Keap1 after H_2_O_2_ stimulation, and thus may inhibit the ubiquitination of Nrf2. Next, anti-Nrf2 immunoprecipitation was analyzed by immunoblot with anti-ubiquitin antibodies for detection of ubiquitin-conjugated Nrf2. The results indicated that PTS attenuated the ubiquitylation of Nrf2. Together, we proved that PTS treatment of myocardial cells leads to the activation of redox-sensitive transcription factor Nrf2, at least in part by interfering with its interaction with Keap1.

### 2.5. PTS Attenuates H_2_O_2_-Induced Cardiomyocyte Apoptosis Depending on Regulation of Nrf2 Activity

**Figure 5:**
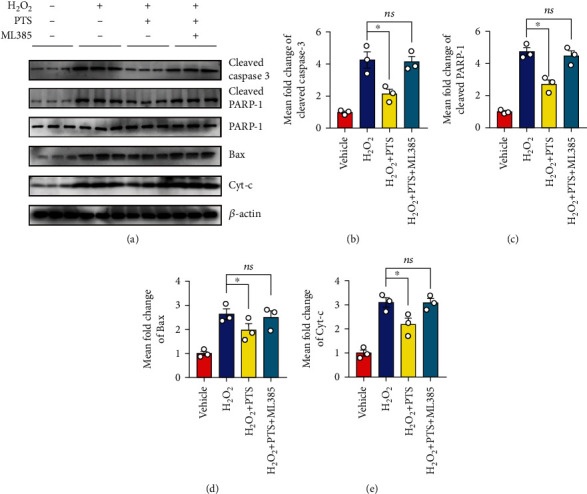
PTS attenuates H_2_O_2_-induced cardiomyocyte apoptosis depending on the regulation of Nrf2 activity. ML385, a direct activator of the Keap1 and Nrf2 interaction, was used to cotreat H9c2 with vehicle or PTS. (a) Western blotting results showed the changes in apoptosis-related proteins in H9c2 cells after Nrf-2 blockade. (b–e) The levels of cleaved caspase-3, cleaved PRAR-1, Bax, and Cyt-c were quantified using ImageJ. Data are expressed as the mean ± SEM (*n* = 3). ^∗^*P* < 0.05, ^∗∗^*P* < 0.01; ns: not significant.

Since the above data suggest that PTS disturbs Keap1/Nrf2 combination to promote the expression of antioxidant elements, further studies are required to determine the relationship between the promoting effects of PTS on nuclear translocation of Nrf2 and inhibiting effects of apoptosis. Therefore, the Nrf2 inhibitor, ML385, was used to examine whether PTS can improve H_2_O_2_-induced cardiomyocyte apoptosis by promoting nuclear translocation of Nrf2. Interestingly, the apoptosis inhibitory effect of PTS was largely blocked in H9c2 cells by pretreatment with ML385, as demonstrated by Western blotting for apoptosis-related proteins (Figures 5(a)–5(e)). Together, these observations indicated that PTS promotes nuclear translocation of Nrf2 and thus reduces H_2_O_2_-induced cardiomyocyte apoptosis.

### 2.6. PTS Attenuates Myocardial Ischemia/Reperfusion Injury (MIRI) in Rats

**Figure 6:**
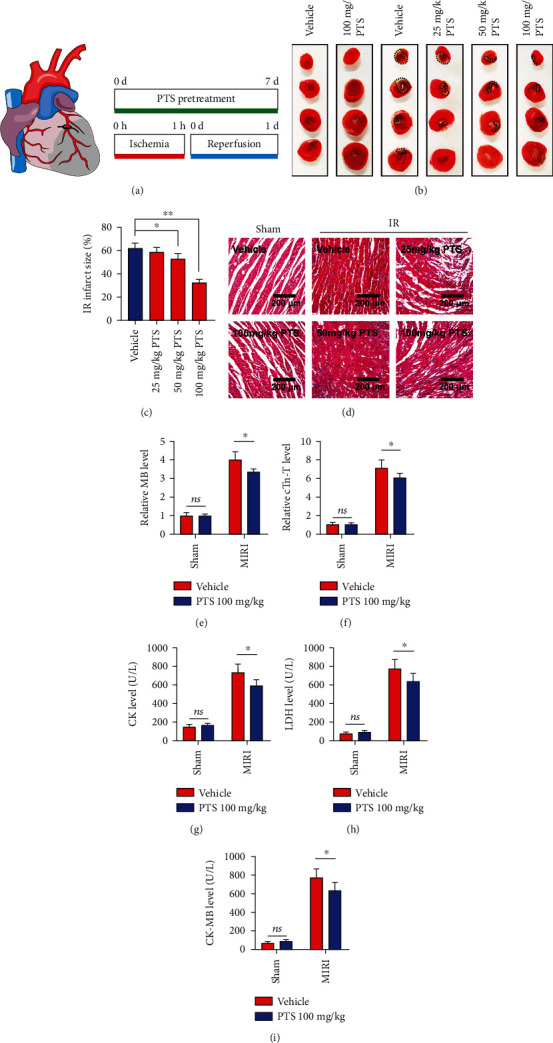
PTS attenuates myocardial ischemia/reperfusion injury (MIRI) in rats. A total of 60 rats were divided into six groups to determine the optimal concentration of PTS to treat MIRI rats, including the I/R group, sham group, sham+PTS group, and I/R+PTS groups (intragastric administration of 25, 50, or 100 mg/kg/d PTS aqueous solution followed by I/R operation). The pretreatment was carried over 7 days. The sham and I/R groups were pretreated with vehicle (water) before the operation. (a) Schematic diagram showing the position of the LAD branch and establishment of the I/R model. (b) Representative images of TTC staining. (c) Infarct size (black dotted circle) was quantified using ImageJ software. (d) Representative images of H&E staining of rat myocardial tissue sections. (e–i) The myocardial injury markers MB, cTn-t, CK, LDH, and CK-MB were quantified using ImageJ. Data are expressed as the mean ± SEM (*n* = 8). ^∗^*P* < 0.05, ^∗∗^*P* < 0.01. ns: not significant. AST: aspartate aminotransferase; CK: creatine kinase; cTn-T: cardiac troponin-T; LDH: lactate dehydrogenase; MB: myoglobin.

Next, we assessed the protective effects of PTS against I/R injury *in vivo*, in a rat model of MIRI involving pretreatment with PTS for 7 days followed by 1 h of ischemia and 1 d of reperfusion (Figure 6(a)). As shown in Figures 6(b) and 6(c), I/R rats showed an increase in myocardial INF compared with the sham group. However, PTS pretreatment significantly decreased INF in comparison with the I/R group. In addition, the I/R group showed obvious myocardial fiber fracture, cellular edema, hemorrhage, necrosis, and neutrophil infiltration. Interestingly, compared with the I/R group, myocardial tissue damage showed gradual recovery with increasing doses of PTS (25, 50, and 100 mg/kg) (Figure 6(d)). I/R significantly induced myocardial injury in rats, as evidenced by the increased serum levels of MB, cTn-T, CK, LDH, and CK-MB. Surprisingly, these abnormal markers were markedly attenuated by pretreatment with PTS (100 mg/kg) (Figures 6(e)–6(i)). Taken together, these observations support the potential value of PTS for the treatment of MIRI.

### 2.7. PTS Ameliorated Myocardial Mitochondrial Apoptosis in MIRI Rats by Regulating Nrf2

**Figure 7:**
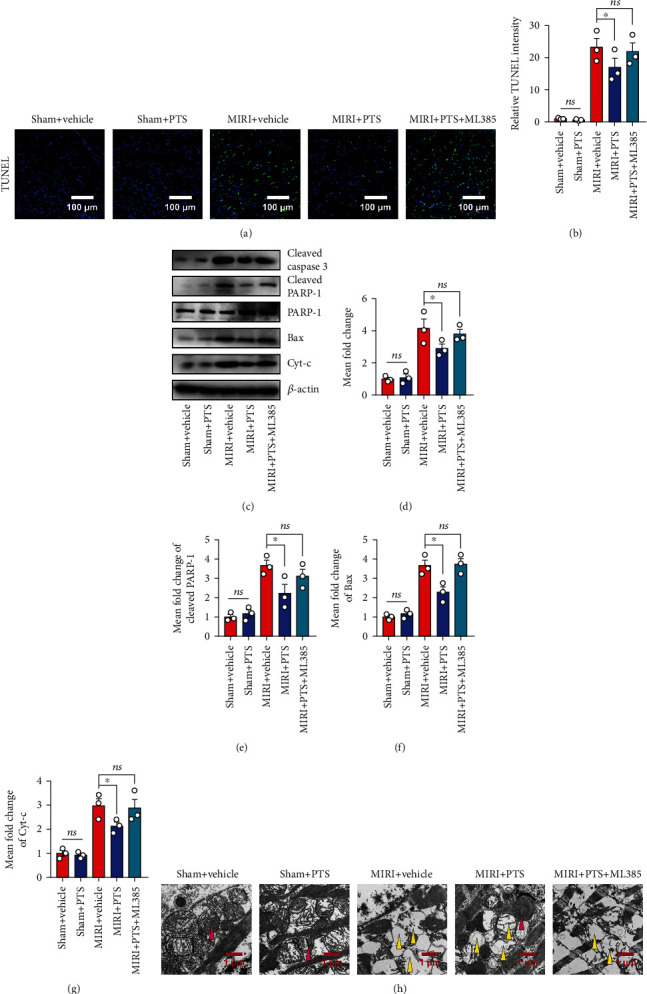
PTS ameliorated myocardial mitochondrial apoptosis in MIRI rats by regulating Nrf-2. A total of 50 rats were divided into five groups to explore the underlying mechanisms including the I/R group, sham group, sham+PTS group, I/R+PTS group, and PTS+ML385 group. (a) Representative TUNEL results of myocardial tissue sections. (b) The relative TUNEL staining intensities of each group were quantified using ImageJ software. (c) Western blotting analysis showed the changes in apoptosis-related proteins in myocardial tissue after Nrf-2 blockade. (d–g) The levels of cleaved caspase-3, cleaved PRAR-1, Bax, and Cyt-c were quantified using ImageJ. (h) The ultrastructure of the rat myocardium was examined by transmission electron microscopy. The red arrow indicates normal myocardial mitochondria, and the yellow arrow indicates damaged mitochondria. Data are expressed as the mean ± SEM (*n* = 3). ^∗^*P* < 0.05, ^∗∗^*P* < 0.01; ns: not significant.

Next, we examined the potential mechanisms underlying the cardioprotective effects of PTS. As the data presented above indicated the antiapoptotic effect of PTS associated with regulation of Nrf2 nuclear translocation, we blocked the activity of Nrf2 and performed I/R surgery according to the same procedure. First, sections of paraffin-embedded rat heart tissue were subjected to TUNEL staining, and the results indicated that I/R markedly induced myocardial apoptosis in rats, as evidenced by the increased TUNEL fluorescence intensity. PTS pretreatment alleviated the cardiomyocyte apoptosis caused by I/R, and this effect was diminished by ML385 administration (5 *μ*M) (Figures 7(a) and 7(b)). Next, the levels of apoptosis-related proteins were analyzed. Western blotting analysis showed that the levels of cleaved caspase-3 and cleaved PARP-1, as well as Cyt-c, were significantly higher, while the level of Bax was significantly lower, in the I/R group compared with the sham and sham+PTS groups. However, the I/R+PTS+ML385 group showed no significant differences in the expression level of these proteins in comparison with the I/R group, indicating that the therapeutic effect of PTS was attenuated by inhibition of Nrf2 (Figures 7(c)–7(g)). Furthermore, representative TEM images of cardiac mitochondria showed rupture of the outer mitochondrial membranes, disappearance of cristae, and even vacuolization in the I/R group. Interestingly, more complete outer mitochondrial membrane structures and cristae, and less vacuolization, were observed in the I/R+PTS group compared with the I/R group, but these differences were inhibited by ML385 administration (Figure 7(h)). Taken together, the above results confirmed that PTS can rescue I/R rat cardiomyocyte mitochondrial apoptosis by regulating Nrf2.

## 3. Discussion

The present study indicated that the cardiomyocyte apoptosis induced by I/R injury was closely related to mitochondrial dysfunction. Interestingly, PTS alleviated redox homeostasis disorders in H9c2 cells and protected against H_2_O_2_ induced-apoptosis in H9c2 cells and NRCMs. Moreover, PTS enhanced mitochondrial activity and mitochondrial membrane potential (ΔΨm), and thus maintained mitochondrial function *in vitro*. We further confirmed that PTS reduced myocardial INF after MIRI in rats *in vivo*, resulting in protection against MIRI. In addition, PTS attenuated apoptosis and maintained the mitochondrial integrity of cardiomyocytes *in vivo*. We also demonstrated that PTS antagonized mitochondria-mediated apoptosis of cardiomyocytes *in vitro* and *in vivo* by regulating Nrf2 nuclear translocation, revealing a potential mechanism for the protective effect of PTS against MIRI.

Oxidative stress occurs when the balance between the generation of ROS and antioxidant defense systems is disturbed [30]. Prolonged ischemia following reperfusion will generate a burst of ROS, including superoxide radical anions, H_2_O_2_, and hydroxyl radicals [31]. These increased ROS levels will further mediate membrane lipid peroxidation and generate malondialdehyde (MDA). The antioxidant enzyme system, including SOD and GSH-Px, is also severely weakened [32]. Therefore, antioxidative stress is considered an important target for treatment of MIRI, and numerous lines of evidence support the application of natural antioxidants to prevent MIRI. Consistent with these previous studies, we demonstrated that H_2_O_2_-induced excessive ROS lessened H9c2 cell viability and also weakened the antioxidant capacity of H9c2 cells, manifested as decreased expression of the antioxidant component, SOD1. However, our data suggested that PTS pretreatment enhanced the antioxidant capacity of cardiomyocytes and reduced the destruction caused by ROS (Figure 1).

Cardiomyocyte apoptosis is an irreversible event caused by MIRI, and reducing apoptosis has proven to be an effective strategy for protection against MIRI [33]. The caspase protease family plays key roles in the mitochondria-mediated apoptotic pathway [34]. The opening of mPTPs leads to inhibition of Bcl-2 activity; Bax is recruited from the cytoplasm to the mitochondria, followed by the release of various apoptotic factors, including Cyt-c, from the mitochondria into the cytoplasm, thereby activating the downstream effector caspases (caspase-3, caspase-6, and caspase-7). In turn, this leads to apoptosis via cleavage of targeted cellular proteins [34, 35]. Our *in vitro* data showed a high apoptosis rate in the H_2_O_2_ group, as revealed by flow cytometry and immunofluorescence analysis (Figure 2). In addition, the levels of proapoptotic proteins, including cleaved caspase-3, Bax, cleaved PARP-1, and Cyt-c, were upregulated in cardiomyocytes (Figure 2). These results were verified in the MIRI animal model, indicating that H_2_O_2_ treatment *in vitro* and I/R injury can uniformly induce mitochondrial apoptosis in cardiomyocytes (Figure 7). Surprisingly, the oxidative stress-induced proapoptotic effect was markedly inhibited by PTS administration *in vitro* and *in vivo*. However, the antiapoptotic effect of PTS was abolished by Nrf2 blockade (Figure 5).

Mitochondria are the main sites of aerobic respiration and are also essential targets of ischemic injury. Previous studies showed that mitochondrial dysfunction also plays a decisive role in MIRI [36, 37]. Briefly, decreased oxygen levels diminish mitochondrial ATP production and induce an increase in intracellular Ca^2+^ levels under conditions of ischemia. During reperfusion, the intracellular Ca^2+^ concentration further increases, causing calcium overload in the cytoplasm and mitochondria. Simultaneously, hypoxia causes dysfunction of the mitochondrial electron transport chain, resulting in increased ROS production. The increases in Ca^2+^ and ROS levels lead to the opening of nonselective, highly conductive permeability transition pores (PTPs) in the mitochondrial inner membrane, as well as changes in mitochondrial membrane permeability. PTP opening further increases mitochondrial Ca^2+^ and ROS levels and stimulates the oxidation of proteins and lipids in mitochondria. Calcium overload and oxidative stress may cause mitochondrial dysfunction, which in turn induces cardiomyocyte apoptosis or necrosis. To further study the role of PTS in the mitochondrial apoptosis induced by oxidative stress, we examined changes in mPTP and mitochondrial membrane potential by live cell staining. As expected, PTS pretreatment inhibited the opening of mPTPs, thereby promoting the recovery of lost ΔΨm in cardiomyocytes (Figure 3). Our *in vivo* results showed that PTS reduced mitochondrial vacuolization after I/R, thus maintaining the integrity of the mitochondrial inner membrane (Figure 7).

Nrf2, a basic leucine zipper transcription factor, plays a critical modulatory role in the cellular redox balance by promoting the transcription of detoxifying and antioxidant genes [38]. Under oxidative conditions, Nrf2 separates from Keap1 in the cytoplasm and translocates to the nucleus, which leads to the transcription of phase II detoxification enzymes and anti-inflammatory proteins. Deregulation of Nrf2 transcriptional activity has been reported in the pathogenesis of multiple diseases, including I/R injury, and the Nrf2/Keap1 axis has emerged as a crucial modulator of cellular homeostasis [38]. In addition, a number of studies supported the beneficial effects of Nrf2 on the regulation of apoptosis [39]. Considering the positive role of PTS in regulating Nrf2 in cerebral I/R [18], we suspect that the antiapoptotic and antioxidant effects of PTS may be mediated by the regulation of Nrf2 activity. Indeed, our *in vitro* experiments showed that oxidative stress reduced the nuclear translocation of Nrf2, and this effect was ameliorated by PTS administration (Figure 4). Taken together, these observations suggested that PTS alleviated MIRI partly via the activation of Nrf2.

Our evidence demonstrated that PTS could induce the nuclear translocation of Nrf2, but the underlying mechanism by which PTS regulation of Nrf2 is not well understood. Recent research has broadly focused on manipulating the Nrf2–Keap1 interaction [40], which modulates Nrf2 activity in therapeutic approaches to MIRI. Therefore, we investigated the effects of PTS on the structure of Nrf2–Keap1. Surprisingly, we found that PTS inhibits the Keap1-mediated reduction of Nrf2 ubiquitination and increment of Nrf2 nuclear translocation, which was proved by the molecular docking and coimmunoprecipitation assay.

Overall, we provided confirmed evidence that the dysregulated oxidative stress promotes mitochondrial apoptosis in cardiomyocytes and the antiapoptotic effect of PTS is at least partly dependent on restored immoderate oxidative stress activity by target Keap1. At present, no drug has been found that can treat MIRI with multiple targets. But there is no doubt that we have identified an effective natural compound in the part of the process of mitochondrial apoptosis and initially revealed its possible mechanism, thus providing a novel strategy for the treatment of MIRI.

## 4. Conclusions

The present study demonstrated that PTS protects against MIRI by coordinating the cellular antioxidant defenses and regulating mitochondrial apoptosis via modulation of Nrf2 activity. Therefore, PTS has therapeutic potential for treating MIRI.

## Data Availability

All data are included in the article.

## Supplementary Materials

Supplementary Materials Supplementary Figure 1: chemical structures and representative HPLC chromatograms of PTS. (A) Chemical structures of R1, Rg1, and Re. (B) HPLC chromatogram of reference compounds. (C) HPLC chromatogram of PTS extract. UV absorbance of the HPLC samples was monitored at 210 nm. Supplementary Figure 2: (A) representative Bax and cleaved caspase-3 immunofluorescence staining of NRCMs. (B) Representative cleaved caspase-3 immunofluorescence staining of H9C2 and quantitative analysis of cleaved caspase-3 expressions were displayed in (C). Data were expressed as mean ± SEM (*n* = 3); ^∗^*P* < 0.05, ^∗∗^*P* < 0.01; ns means no significant. Supplementary Table 1: antibodies used.
