# Supplementary material for: Pretreatment with Panaxatriol Saponin Attenuates Mitochondrial Apoptosis and Oxidative Stress to Facilitate Treatment of Myocardial Ischemia-Reperfusion Injury via the Regulation of Keap1/Nrf2 Activity
Source: Oxid Med Cell Longev. 2022 May 27;2022:9626703. doi: 10.1155/2022/9626703 (PMC9166985; doi:10.1155/2022/9626703)
Supplement: Supplementary Materials — Supplementary Figure 1: chemical structures and representative HPLC chromatograms of PTS. (A) Chemical structures of R1, Rg1, and Re. (B) HPLC chromatogram of reference compounds. (C) HPLC chromatogram of PTS extract. UV absorbance of the HPLC samples was monitored at 210 nm. Supplementary Figure 2: (A) representative Bax and cleaved caspase-3 immunofluorescence staining of NRCMs. (B) Representative cleaved caspase-3 immunofluorescence staining of H9C2 and quantitative analysis of cleaved caspase-3 expressions were displayed in (C). Data were expressed as mean ± SEM (n = 3); ∗P < 0.05, ∗∗P < 0.01; ns means no significant. Supplementary Table 1: antibodies used. [file 9626703.f1.zip › Supplementary Materials.pdf]

# *Supplementary Material*

## Supplementary Figures

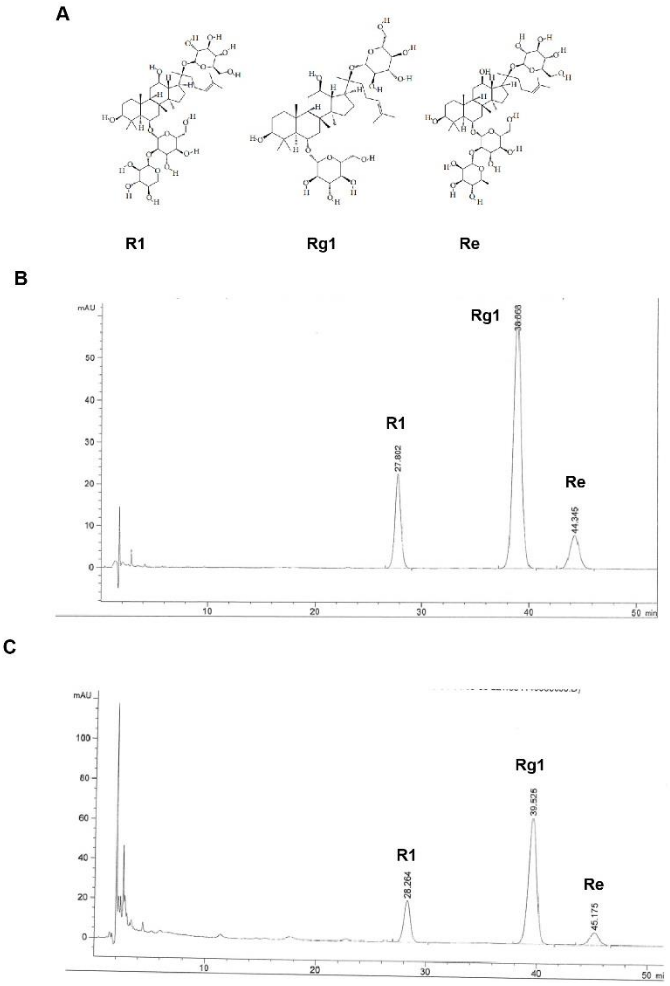


**Supplementary Figure 1.** Chemical structures and representative HPLC chromatograms of PTS. (A) Chemical structures of R1, Rg1, and Re. (B) HPLC chromatogram of reference compounds. (C) HPLC chromatogram of PTS extract. UV absorbance of the HPLC samples was monitored at 210 nm.

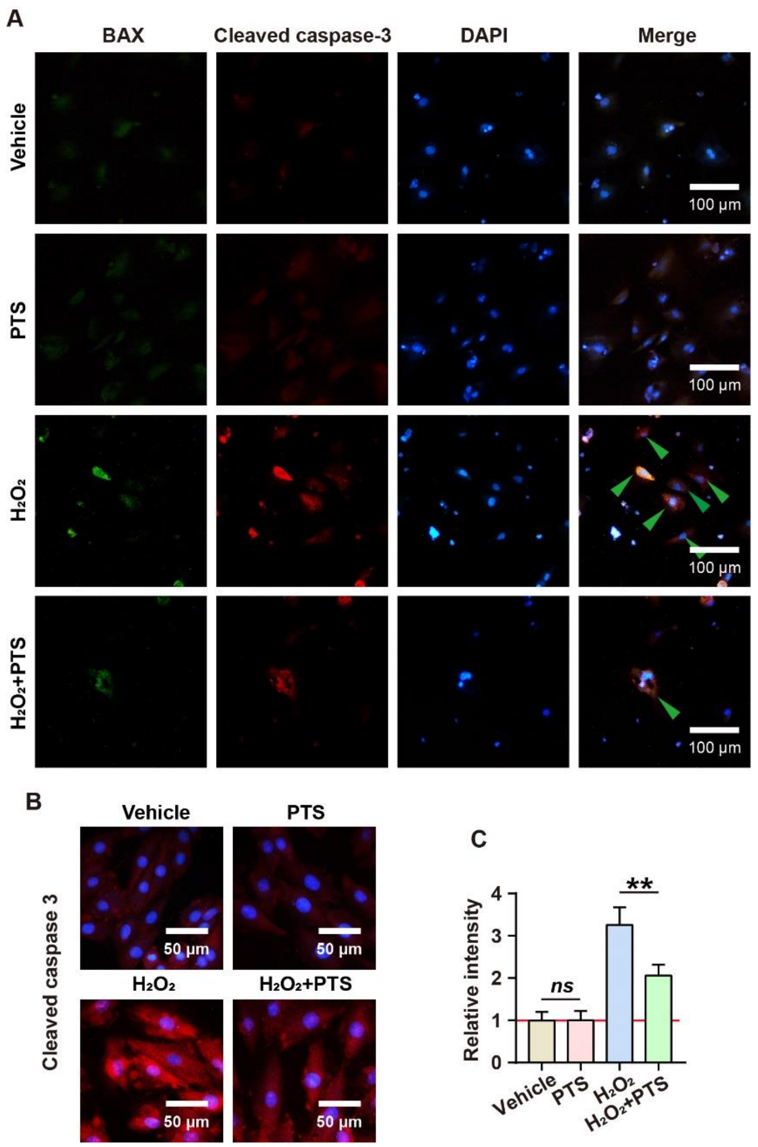


**Supplementary Figure 2.** (A) Representative Bax and cleaved caspase 3 immunofluorescence staining of NRCMs. (B) Representative cleaved caspase 3 immunofluorescence staining of H9C2 and quantitative analysis of cleaved caspase 3 expression was displayed in (C). Data were expressed as mean ± SEM, n=3, **P* < 0.05, ***P* < 0.01, ns means no significant.

**Supplementary Tables**

**Supplementary Table 1. Antibodies used**

| Antibody | Catalog | Company | Application | Dilution |
|---|---|---|---|---|
| SOD1 | 10269-1-AP | Proteintech | IF/WB | 1:200/1:1000 |
| Cleaved caspase-3 | 19677-1-AP | Proteintech | IF/WB | 1:200/1:1000 |
| Cleaved PARP-1 | 13371-1-AP | Proteintech | WB | 1:1000 |
| Bax | 60267-1-lg | Proteintech | IF/WB | 1:200/1:1000 |
| Cyt-c | 10993-1-AP | Proteintech | WB | 1:1000 |
| Nrf2 | 16396-1-AP | Proteintech | IF/WB | 1:200/1:1000 |
| | 66504-1-Ig | Proteintech | IP | 10 μg |
| Keap1 | 60027-1-Ig | Proteintech | WB | 1:1000 |
| | 10503-2-AP | Proteintech | IP | 10 μg |
| Ubiquitin | 10201-2-AP | Proteintech | WB | 1:1000 |
| HO1 | 10701-1-AP | Proteintech | IF/WB | 1:200/1:1000 |
| β-actin | HRP-60008 | Proteintech | WB | 1:5000 |
